# Actionability of commercial laboratory sequencing panels for newborn screening and the importance of transparency for parental decision-making

**DOI:** 10.1186/s13073-021-00867-1

**Published:** 2021-03-29

**Authors:** Daniela M. DeCristo, Laura V. Milko, Julianne M. O’Daniel, Ann Katherine M. Foreman, Lonna F. Mollison, Bradford C. Powell, Cynthia M. Powell, Jonathan S. Berg

**Affiliations:** 1 Department of Genetics, University of North Carolina at Chapel Hill School of Medicine, Chapel Hill, NC 27599 USA; 2 Department of Pediatrics, Division of Genetics and Metabolism, University of North Carolina at Chapel Hill School of Medicine, Chapel Hill, NC 27599 USA

## Abstract

**Background:**

Newborn screening aims to identify individual patients who could benefit from early management, treatment, and/or surveillance practices. As sequencing technologies have progressed and we move into the era of precision medicine, genomic sequencing has been introduced to this area with the hopes of detecting variants related to a vastly expanded number of conditions. Though implementation of genomic sequencing for newborn screening in public health and clinical settings is limited, commercial laboratories have begun to offer genomic screening panels for neonates.

**Methods:**

We examined genes listed on four commercial laboratory genomic screening panels for neonates and assessed their clinical actionability using an established age-based semi-quantitative metric to categorize them. We identified genes that were included on multiple panels or distinct between panels.

**Results:**

Three hundred and nine genes appeared on one or more commercial panels: 74 (23.9%) genes were included in all four commercial panels, 45 (14.6%) were on only three panels, 76 (24.6%) were on only two panels, and 114 (36.9%) genes were listed on only one of the four panels. Eighty-two genes (26.5%) listed on one or more panels were assessed by our method to be inappropriate for newborn screening and to require additional parental decision-making. Conversely, 249 genes that we previously identified as being highly actionable were not listed on any of the four commercial laboratory genomic screening panels.

**Conclusions:**

Commercial neonatal genomic screening panels have heterogeneous content and may contain some conditions with lower actionability than would be expected for public health newborn screening; conversely, some conditions with higher actionability may be omitted from these panels. The lack of transparency about how conditions are selected suggests a need for greater detail about panel content in order for parents to make informed decisions. The nuanced activity of gene list selection for genomic screening should be iteratively refined with evidence-based approaches to provide maximal benefit and minimal harm to newborns.

**Supplementary Information:**

The online version contains supplementary material available at 10.1186/s13073-021-00867-1.

## Background

Genomic sequencing applied to newborn screening (NBS) can potentially improve health outcomes by detecting a large number of conditions, yet there are particular challenges surrounding the implementation of genetic testing in a vulnerable population such as healthy neonates [1–3]. The setting in which genomic screening of neonates is conducted (public health NBS, pediatric well-child care, commercial consumer-directed sequencing) is expected to have a substantial influence on policies, guidelines, expectations, and goals of such screening [4]. For example, for population-based NBS, recommendations typically emphasize equity in accessibility and availability of testing. In clinical medicine, ethical issues of genetic testing in the pediatric population have been outlined, weighing the benefit to the child and family [1]. Expanded genomic screening tests offered through commercial laboratories are generally intended for the purpose of providing information about a broader number of conditions than traditional NBS, but are still intended for use in an asymptomatic child.

The benefits and harms of providing additional predictive genomic information in healthy newborns, outside of state-directed programs, are not well understood [5, 6]. Recommendations have been made to protect the future decision-making capacity of children, and professional guidelines and expert opinion emphasize the availability of preventative management practices for conditions that are tested [5, 7, 8]. However, commercial laboratory policies for testing in the healthy pediatric population are inconsistent, can be opaque, and may sidestep critical issues related to genetic testing in minors altogether [9]. Therefore, NBS through commercial laboratories may be juxtaposed with public health efforts to coordinate equitable and acceptable screening practices that benefit all newborns.

The availability of both clinical and commercial genomic sequencing has changed the screening landscape [4]. As public interest in precision medicine and human genetics has increased, commercial laboratories have stepped in to provide other genetic testing avenues using advanced sequencing technologies at competitive rates and partnering with healthcare professionals and institutions [6, 10]. These technologies are rapidly evolving, and the number of gene-disease associations continues to expand while evidence for the clinical validity and clinical actionability of that information often lags behind [11]. Consequently, although the number of genes on sequencing panels has grown to capture these new findings, it is unclear whether those offerings represent information that would be consistent with public health NBS and pediatric genetic testing recommendations [12, 13]. Thus, although expanded genomic screening products offered through commercial laboratories are generally intended for the purpose of providing information about a broader number of conditions in an asymptomatic child, the harms and benefits of providing additional information outside of state-directed programs are not well understood. The programmatic infrastructure for follow-up and management (such as state contracts with metabolic specialist clinics) may not exist for many of the conditions, thus limiting access to expert care for those who screen positive [5, 6].

Commercially offered supplemental genomic screening panels have not been systematically examined and compared, and the clinical actionability of genes on these panels or the lab-based inclusion criteria are not publicly available for reference. Comparisons of genes across commercial panels can elucidate similarities and differences between various strategies for tailoring genetic testing to a pediatric population. This study aimed to identify areas of consensus and discordance across panels by assessing the clinical actionability of genes included on commercial laboratory genomic screening sequencing panels, and their associated conditions, to evaluate the current trajectory of commercially offered genomic screening in newborns.

## Methods

### Identifying commercial laboratory neonatal genomic screening sequencing panels

Commercial laboratory sequencing panels for supplemental or expanded NBS were identified manually via the NIH Genetic Testing Registry (GTR) and Google search [14]. Four panels had gene lists that were publicly available and were obtained for analysis: Baby Genes Supplemental Newborn Screening [15] (109 genes), Sema4 Natalis [16] (166 genes), Fulgent Newborn Genetic Analysis NGS Panel [17] (255 genes), and PerkinElmer Expanded Newborn Screening and Gene Sequencing Panel [18] (275 genes).

### Defining the gene-disease association

When the genes on panels were listed with an associated disease, those gene-disease pairs were curated or matched with the score assigned previously using an established age-based semi-quantitative (ASQM) metric [19]. When there was no phenotype provided, the gene was matched to gene-disease associations that had been scored, and the highest scoring (i.e., most actionable) phenotype associated with the gene was used for the comparison. Genes with multiple phenotypes that were differentially scored and categorized using the ASQM and appeared on commercial laboratory panels were selected for further evaluation.

### Scoring medical actionability

Actionability scores were based on five criteria measured in a previously published semi-quantitative metric (SQM): severity of disease, likelihood of disease presentation, efficacy of intervention, acceptability of intervention, and the knowledgebase or amount of evidence available to score the prior four criteria [20]. Scores of 0 to 3 were assigned for each category, with a total maximum SQM score of 15, where higher scores indicate a greater degree of severity, likelihood, efficacy, acceptability, and/or knowledge. Based on the SQM score, the typical age of onset of the condition, and age of onset of the intervention, pairs were placed in four different categories of screening results [19] for use in a clinical trial that explored the application of exome sequencing in newborns [21]:

- Category 1—Conditions with childhood onset that are highly actionable (which were deemed eligible for result disclosure in newborns undergoing exome sequencing as part of the clinical trial)
- Category 2—Conditions with childhood onset but lower actionability (which were reserved for parental decision making in the clinical trial)
- Category 3—Conditions with adult onset and high actionability (which were reserved for parental decision making in the clinical trial)
- Category 4—Conditions with adult onset and low actionability (which were not eligible to be disclosed in healthy newborns in the clinical trial)

As described previously, childhood onset gene-disease pairs scoring 12 or higher were considered “actionable” while those scoring 9, 10, or 11 were discussed by the scoring committee until a consensus was reached about whether the condition qualifies for Category 1 or Category 2. Gene-disease pairs with controversial evidence or onset before birth were excluded. Individual scores and categorizations were previously published for 822 gene-disease pairs. Additional gene-disease pairs that were listed on the commercial panels were scored and categorized for this study using the previously published method [19].

### Analyzing gene-disease scores across commercial laboratory NBS panels

Gene-disease pairs on commercial laboratory panels were manually matched to entries previously curated using a REDCap database and scored using the ASQM. Genes classified as “Category A” as part of the BabySeq project [22] were also included in the current analysis. Prism GraphPad was used for statistical analyses and figures. Kruskal–Wallis and Mann–Whitney *U* tests were performed to compare ASQM scores between various proposed neonatal genomic screening panels using a significance level of 0.05.

## Results

### Commercial laboratory neonatal genomic screening panels intersect

**Fig. 1:**
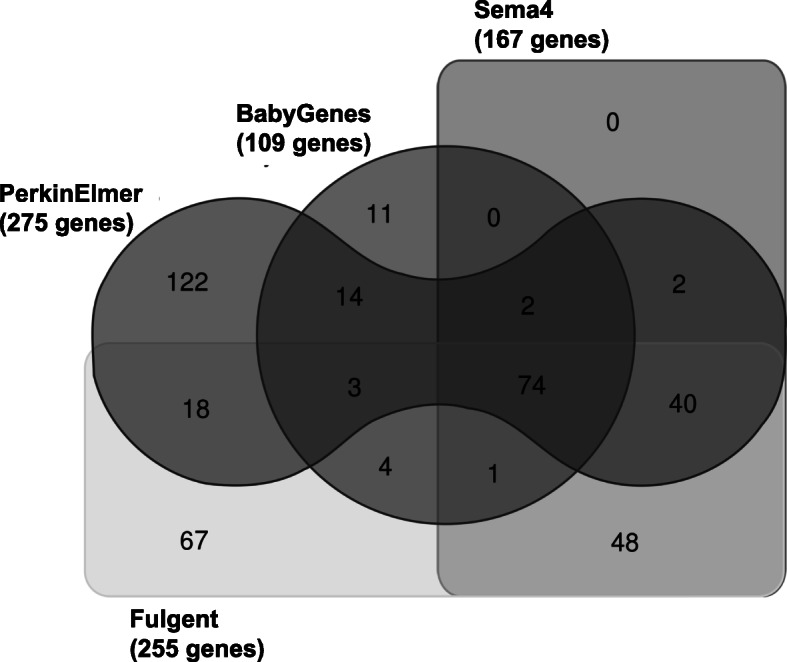
Comparison of genes on commercial laboratory NBS panels. Venn diagram summarizing all of the overlapping or distinct genes from BabyGenes Supplemental Newborn Screening, Sema4 Natalis, Fulgent Newborn Genetic Analysis NGS Panel, and PerkinElmer Expanded Newborn Screening and Gene Sequencing Panel using a free Ghent University Bioinformatics and Evolutionary Genomics software tool [23]. A single gene entry for *HBA1*/*HBA2* on Sema4 Natalis was split for comparison, and the total number of genes on the panel changed from 166 to 167

Four commercial laboratory next-generation sequencing (NGS) NBS gene panels were identified: Baby Genes Supplemental Newborn Screening (109 genes), Sema4 Natalis (166 genes), Fulgent Newborn Genetic Analysis NGS Panel (255 genes), and PerkinElmer Expanded Newborn Screening and Gene Sequencing Panel (275 genes) [15–18]. All labs were listed on the GTR except for Fulgent. Genes *HBA1* and *HBA2* were considered separately for analysis although Sema4 Natalis grouped them together. A total of 74 genes were included in all four commercial panels, 45 appeared on three panels, 76 appeared on two panels, and 114 genes were listed on only one of the four panels (Fig. 1). Comparing the number of genes that were only included in one of the four panels, 11 out of 109 genes (10.1%) were unique to the Baby Genes Supplemental Newborn Screening panel, 122 out of 275 genes (44.4%) were unique to the PerkinElmer Expanded Newborn Screening and Gene Sequencing Panel, and 67 out of 255 genes (26.3%) were unique to the Fulgent Newborn Genetic Analysis NGS Panel. Sema4 Natalis did not include any genes that were not present on at least one of the other three commercial NBS panels. Considering previously published lists of genes that may be appropriate for screening in newborns, a total of 61 genes were included on all of the commercial panels as well as the BabySeq Category A and ASQM Category 1 panels. Thus, there is considerable agreement among different groups regarding a subset of gene-disease pairs that could be offered as a consensus expanded newborn screening panel (Additional file 1: Table S1).

### Variability of actionability score distributions: commercial laboratory panels

**Fig. 2:**
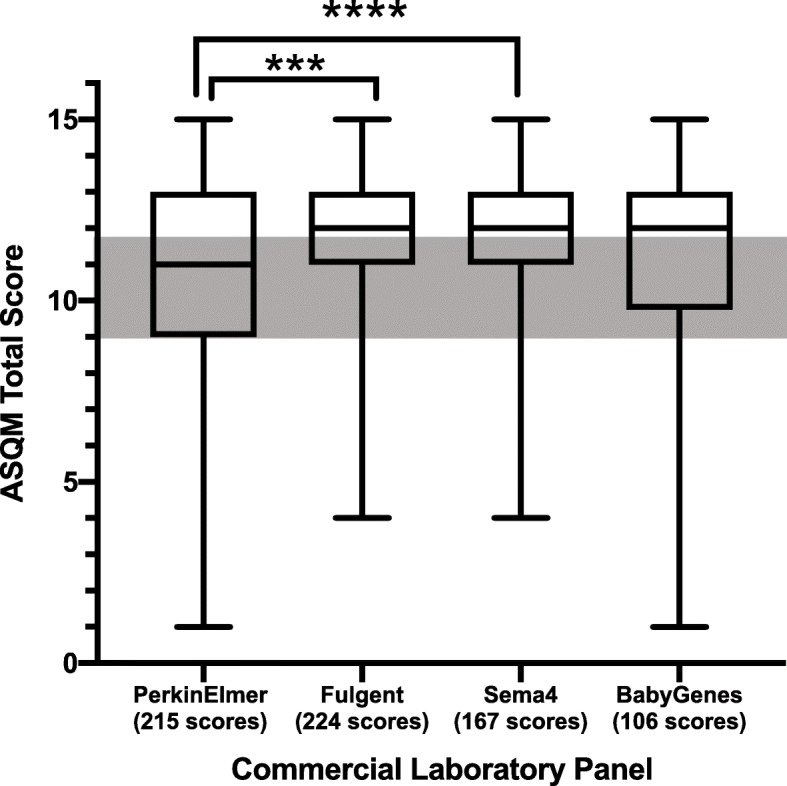
Comparison of ASQM total scores for genes on commercial laboratory NBS panels. Box and whisker plot of ASQM score distributions for 215 genes on PerkinElmer’s panel, 224 scores on Fulgent’s panel, 167 genes on Sema4’s panel, and 106 genes on BabyGenes’ panel. All genes that were scored using the ASQM were used for comparison, regardless of category. The gray box represents the area where genes-disease pairs scoring 9, 10, or 11 could not be automatically categorized and necessitated further discussion by the scoring committee prior to final categorization. Distributions varied significantly across all panels by Kruskal–Wallis test (*p* < 0.0001), and paired Mann–Whitney *U* tests identified significant differences between PerkinElmer and Fulgent panels' ASQM gene-disease pair scores (***p = 0.0001) and between PerkinElmer and Sema4 panels' ASQM gene-disease pair scores (****p < 0.0001)

Next, we examined the ASQM scores for gene-disease pairs included in each of the commercial panels. We scored 88 additional gene-disease pairs (Additional file 1: Table S2), combined them with the scores of 822 pairs published previously, and matched these scores to 215 genes on the PerkinElmer panel, 224 genes on the Fulgent panel, 167 genes on the Sema4 panel, and 106 genes on the Baby Genes panel. The averages and ranges of scores are shown in Table 1. ASQM scores across all panels showed differences in overall distribution (Kruskal–Wallis *p* < 0.0001), with two significantly different distributions of ranks identified between PerkinElmer and Fulgent panels (*p* = 0.0001) and PerkinElmer and Sema4 panels (*p* < 0.0001) through nonparametric pairwise comparisons (Fig. 2).

**Table 1:** Averages and ranges for commercial laboratory, ASQM, and BabySeq NBS panels

	ASQM scores			
NBS Panel	Mean	Median	Minimum	Maximum
BabyGenes	11.019	12	1	15
Sema4	11.772	12	4	15
Fulgent	11.710	12	4	15
PerkinElmer	10.628	11	1	15
BabySeq “Category A”	11.047	12	1	15
ASQM “Category 1”	11.658	12	9	15

Mean, median, minimum and maximum ASQM scores for PerkinElmer (*n* = 215), Fulgent (*n* = 224), Sema4 (*n* = 167), BabyGenes (*n* = 106), BabySeq “Category A” (*n* = 358), and ASQM “Category 1” (*n* = 551) panels

### Variability of actionability score distributions: overlapping genes

**Fig. 3:**
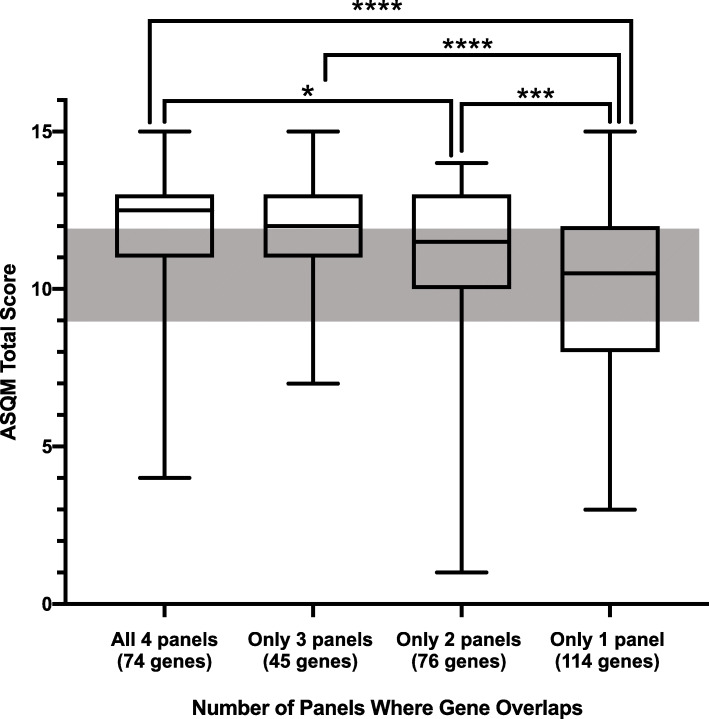
Comparison of ASQM scores of overlapping or distinct genes on commercial panels. Box and whisker plot of ASQM scores of genes that appeared on all four commercial laboratory NBS panels, only three commercial panels, only two commercial panels, and only one commercial panel. The gray box represents the area where genes-disease pairs scoring 9, 10, or 11 could not be automatically categorized and necessitated further discussion by the scoring committee prior to final categorization. Distributions varied significantly across all panels by Kruskal–Wallis test (*p* < 0.0001), and paired Mann–Whitney *U* tests identified significant differences in three out of four comparisons between scores of genes on all four panels versus only two panels (*p = 0.429), scores of genes on all four panels versus only one panel (****p < 0.0001), scores of genes on three panels versus only one panel (****p < 0.0001), and scores of genes on two panels versus only one panel (***p = 0.0001)

Scores varied significantly between the groups of genes that were included on all four panels, as compared to only three panels, only two panels, and only one panel (*p* < 0.0001). There was a general trend for higher median actionability scores among the lists of genes that were included on three or four panels as opposed to only one or two panels. Statistically significant differences in distributions of ranks were observed in four out of six nonparametric pairwise comparisons (Fig. 3).

### Multiple phenotypic associations lead to distinct gene-disease scores and panel categorization

**Fig. 4:**
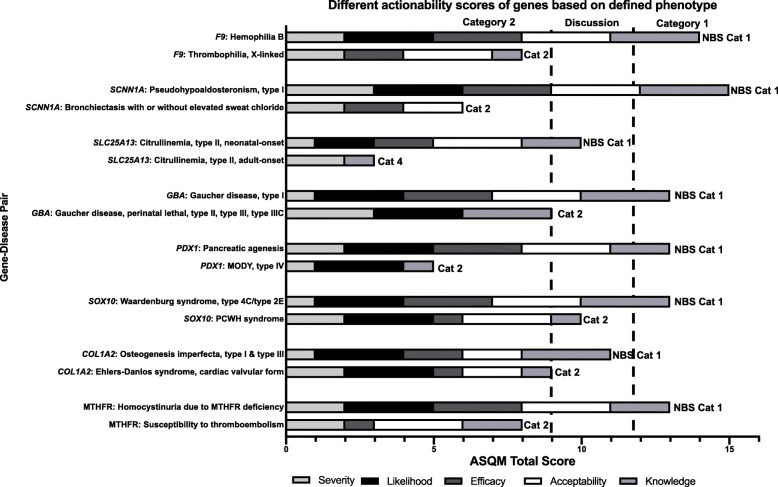
Examples of different actionability scores based on defined phenotype. Bar graph of ASQM scores for eight genes with conditions that were separately reviewed by the scoring committee depicting individual criterion scores for the severity of disease, likelihood of presentation or penetrance, efficacy and acceptability of intervention, and knowledge base. The area between the vertical dashed lines represents gene-disease pairs scoring 9, 10, and 11 that underwent an additional round of discussion and review prior to categorization. Genes associated with early-onset conditions that scored 8 or below were automatically placed in ASQM Category 2 and pairs scoring 12 or greater were placed in ASQM Category 1. Assigned ASQM categories are labeled at the end of each bar for every gene-disease pair: *NBS Cat 1* for Category 1 with early-onset and high actionability, *Cat 2* for Category 2 with early-onset but lower or no actionability, and *Cat 4* for Category 4 with adult-onset and lower or no actionability

There were 81 genes associated with multiple gene-disease associations (based on either inheritance pattern, phenotypic severity, or molecular mechanism), each of which were reviewed separately using the ASQM framework, resulting in different total scores. Of these, 35 genes appeared on one or more commercial laboratory NBS panels. Figure 4 shows examples of eight genes with multiple disease associations resulting in differential ASQM scoring and categorization. The *F9* gene appeared on Sema4’s and Fulgent’s panels; *SCNN1A*, *SOX10*, and *COL1A2* appeared on Fulgent’s panel; *PDX1* appeared on PerkinElmer’s panel; *GBA* and *MTHFR* appeared on BabyGenes’ panel; and *SLC25A13* appeared on all four commercial laboratory panels. Sema4 and BabyGenes specifically referenced the disease associated with each gene that was listed on their panels. Factor IX deficiency or hemophilia B was listed for *F9* on Sema4 Natalis. Gaucher disease was listed for *GBA* and homocystinuria was listed for *MTHFR* on BabyGenes Supplementary Newborn Screening Panel. Using the ASQM, these three gene-disease pairs were scored as having higher actionability out of the two gene-disease associations that were considered for *F9*, *GBA*, and *MTHFR*. Thrombophilia associated with variants in *F9*, for which the outcome considered by our group was deep vein thrombosis, was given a lower gene-disease pair score than hemophilia B associated with *F9* variants, due to limited evidence about penetrance. For *GBA*, Gaucher disease type 1 was evaluated separately from all other types that have severe neurodegenerative phenotypes; enzyme replacement therapy for Gaucher disease type 1 was considered effective (resulting in higher actionability), whereas it was considered less effective for other disease types. *MTHFR* disease associations were scored separately for homocystinuria due to MTHFR deficiency and susceptibility to thromboembolism. The outcome considered for both gene-disease pairs was thrombosis. Likelihood of disease presentation or penetrance was given a score of 0 and efficacy of intervention was given a score of 1 for susceptibility to thromboembolism, whereas scores of 3 were assigned to both likelihood and efficacy criteria for the *MTHFR* and homocystinuria gene-disease pair. These distinctions, while seemingly subtle, require the laboratory to carefully consider the phenotypic association for any given variant(s) in these genes when deciding which results to report, and how to communicate the clinical significance and actionability of those results.

### Discrepancy between commercial laboratory gene panels and ASQM categorization

In our previous work, conditions having higher actionability and childhood onset (Category 1) were considered appropriate for disclosure to all participants in our clinical trial, which examined the potential use of exome sequencing for extended newborn screening [19, 21]. Childhood onset conditions with lower actionability (Category 2) and adult-onset actionable conditions (Category 3) were deemed to require parental decision-making. Adult-onset conditions with lower actionability (Category 4) were not returned to any participants. We examined the distribution of the gene-disease pairs included in the commercial supplemental NBS panels with respect to these categories. Of the 74 gene-disease pairs listed on all four commercial panels (Additional file 1, Table S1), the majority were designated as Category 1, but 9 of them (12.2%) were categorized as having lower actionability and were placed in Category 2. Of the 309 genes that appeared on one or more commercial panels, 82 genes (26.5%) were not included in our Category 1 list. Of these, 77 genes were considered childhood onset with low actionability and placed in Category 2 (Additional file 1, Table S3). Additionally, 5 genes (*NF2* on Fulgent and *SERPINA1*, *HGD*, *NAGA*, *PRNP* on PerkinElmer) were defined as actionable but having an age of onset in adulthood by our group (Table 2). This may be due in part to variable expressivity of some conditions in terms of age of onset, or different expectations about when clinical interventions (surveillance or preventive measures) might be indicated in a person with a disease-associated genotype.

**Table 2:** Genes on commercial laboratory NBS panels considered adult-onset by ASQM

Gene	OMIM phenotype	Inheritance	Outcome considered	Age of onset	Intervention considered	Age of intervention implementation	Severity	Likelihood	Efficacy	Acceptability	Knowledge	Total	ASQM Category
*NF2*	Neurofibromatosis type 2	AD	Acoustic neuroma and meningiomas	Adulthood	Referral to specialists for surveillance and early management	Adolescent	1	3	1	3	3	11	**3**
*SERPINA1*	Emphysema-cirrhosis, due to AAT deficiency	AR	Syndromic manifestations	Adulthood	Referral to specialists for surveillance and early management	Adulthood	2	3	1	3	3	12	**3**
*HGD*	Alkaptonuria	AR	Arthritis	Adulthood	Referral to specialists for surveillance and supportive care	Adulthood	1	3	0	0	3	7	**4**
*NAGA*	Kanzaki disease (Schindler’s Disease type II)	AR	Syndromic manifestations	Adolescent/Adult	Referral to specialists for surveillance and supportive care	N/A	1	3	0	0	1	5	**4**
*PRNP*	Creutzfeldt-Jakob disease / Huntington disease-like 1 / Prion disease with protracted course/Cerebral amyloid angiopathy, PRNPrelated/Gerstmann-Straussler disease / Insomnia, fatal familial / {Kuru, susceptibility to}	AD	Progressive neurodegeneration	Adulthood	Referral to specialists for surveillance and supportive management	N/A	2	3	0	0	2	7	**4**

Five genes listed on commercial laboratory NBS panels were defined as being associated with an adult-onset condition and categorized into ASQM Category 3 or ASQM Category 4 based on actionability scoring. *NF2* was listed on Fulgent’s panel and *SERPINA1*, *HGD*, *NAGA*, *PRNP* were all found on PerkinElmer’s panel. Phenotypic association, inheritance, outcome considered for scoring, age of onset, intervention considered for scoring, individual scores for the five ASQM criteria (severity of disease, likelihood of presentation, efficacy and acceptability of intervention, and knowledge base), ASQM total score, and category indicate what was reviewed and decided upon by the scoring committee

Conversely, there were 249 genes previously assessed by our group and placed into Category 1 that were not listed on any of the four commercial lab NBS panels (Additional file 1, Table S4). Of these, 110 genes (44.2%) scored 12 or higher and were therefore considered highly actionable. Examples of genes in Category 1 that scored 12 or higher and were not listed on commercial laboratory panels include: *F8* associated with hemophilia A and other hemophilia-associated genes, *VWF* associated with von Willebrand disease type 3, *MITF* associated with Waardenburg syndrome type 2A, *BAG3* associated with cardiomyopathy and other cardiomyopathy-associated genes, *TGFB3* associated with Loeys-Dietz syndrome, *PIK3CD* associated with immunodeficiency and other immunodeficiency-associated genes, and *RET* associated with multiple endocrine neoplasia type 2 and medullary thyroid carcinoma.

## Discussion

NBS was implemented in the USA as a public health intervention because the clear benefit of early identification and treatment of certain conditions outweighs the risk of harm, thus leading to a situation in which screening is performed in virtually every individual, regardless of where they are born, without extensive parental decision-making. Procedures are in place for state laboratories to efficiently process and report results to primary care physicians and to alert specialists to positive findings. Management programs for the most commonly detected conditions are well established and (typically) funded by the states to ensure that anyone who is identified through NBS can be followed up for subsequent diagnostic testing and treatment. A process exists for identification of new conditions that could be added to public health screening programs and for establishing recommended uniform screening panels [24]. The advantages of this system from a public health system standpoint are that the benefits of screening, as well as the costs, are shared broadly across society. When new technology is introduced that could increase the number of conditions screened for, a structured and transparent decision-making process is required to determine how best to implement that new technology in a public health setting [25].

Conversely, commercially offered supplemental NBS options have preceded widespread public health adoption of newborn genomic screening. Inherent conflicts of interest within the commercial sector, and vaguely defined expanded screening offerings, may diverge significantly with traditional consensus about inclusion criteria for public health interventions [26]. The 249 genes that were categorized by our group as highly actionable, but not included on any of the four commercial laboratory panels, demonstrate the importance of a systematic approach such as the ASQM for decision-making about conditions to include on genomic screening panels. However, while appraisal of clinical actionability to inform these decisions is an important starting point, other factors may be relevant to decisions regarding population screening of apparently healthy neonates and children. These factors include the prevalence of the condition, the clinical performance of a next-generation sequencing assay for a given gene, the threshold above which conditions are perceived as being sufficiently “clinically actionable” to include, and the cost and cost-effectiveness of downstream medical interventions that would be recommended for any given condition. It is unknown whether any of these considerations were taken into account by the commercial laboratories when designing their panels.

Genomic sequencing (either with targeted panels or genome-scale sequencing) introduces a tremendous opportunity to increase the conditions that can be screened, yet creates tension between societal welfare, parental responsibilities, and commercial interests. The blurring of the lines between a clinically oriented test that is marketed to parents, but ordered by physicians, for the purpose of screening for rare disorders outside of an organized public health infrastructure, necessitates an examination of what is actually being offered and how it might impact the health and well-being of the children who are undergoing testing. In the USA, for example, each state currently establishes procedures for follow-up of positive NBS findings by subspecialists with defined management plans; addition of a broad range of conditions, especially those with incomplete penetrance, may require additional guidance to primary care providers with regard to how positive findings should be disclosed and/or followed up. Given the marketing of this test to parents as a form of NBS, it is important that parents are made aware of the degree of actionability of findings that they may learn about their child, so as not to create a false impression that the findings are necessarily comparable to other conditions evaluated in traditional NBS. It is perhaps predictable that there would be a number of genes on these panels with varying degrees of actionability, since a parent who seeks out supplemental screening may be interested in all types of information that would be clinically relevant to their child. It is critical, however, that these be fully informed choices, which may be at odds with the commercial goals of laboratories to sell the most tests.

The need for transparency in a public health screening setting has been demonstrated in assessments of parental knowledge and values surrounding newborn screening. Transparent information-giving may facilitate decision-making by openly presenting potential benefits and harms of testing and engendering trust [27–29]. Decision aids have been developed to integrate parental values and opinions into the educational content they are provided [30, 31]. However, the effectiveness of these tools is dependent on the information available regarding the nature of the potential information to be learned, which will vary based on the content of the panel. For providers, this challenge revolves around the ability to succinctly summarize the content and potential results that could be returned. For parents who expect information that will help them protect their child’s health, heterogeneity with regard to the clinical actionability of the information that might be revealed could result in some parents receiving information that is surprising and potentially disturbing due to the inclusion of conditions with little or no actionability in newborns and children.

### Commercial laboratory neonatal genomic screening panels intersect

When we searched for genetic testing panels that were being marketed for supplemental NBS, we identified four such offerings. This was not intended to be an exhaustive or comprehensively updated search, and the available options may change rapidly. In fact, during the preparation of this manuscript, Baby Genes was acquired by ArcherDx [32] and the list of “over 100 genes covering more than 72 clinically-actionable, inherited conditions as well as pre-defined carrier screening tests that include full-gene sequencing for Cystic Fibrosis (CF), Spinal Muscular Atrophy (SMA) and Fragile X Syndrome” included in the supplemental neonatal genomic screening panel is no longer publicly available.

Nevertheless, there was substantial overlap between the four commercial NBS panels, with 74 genes being included on every panel and 121 genes included on two or three of the panels. Interestingly, all the genes on Sema4 Natalis appeared on at least one other NBS commercial panel although it was not the smallest panel of the group. Of interest are the 114 genes that are included on only one panel. While having more genes on a panel may give the impression of a more “comprehensive” testing option, the old adage “more is not necessarily better” is important to consider for this situation as in many other areas of medicine [33]. The key question is whether the additional content included on supplemental newborn screening panels is expected to have the same level of actionability as evidence-based NBS conditions [34–36]. Genes correlated to conditions included in traditional public health newborn screening programs might be expected to be listed, but this becomes complicated given that conditions identified through phenotypic screens, such as hearing loss and cyanotic heart disease, could have a very broad range of both genetic and non-genetic etiologies [37, 38]. It remains to be explored whether it would be more informative for neonatal genomic sequencing tests to cover conditions that are not addressed with current newborn screening and thereby extend the range of conditions that can be detected.

### Variability of actionability score distributions across different panels

To examine the consistency between the commercially available testing panels, we applied the SQM which we have previously validated as a measure of clinical actionability by comparison to the American College of Medical Genetics and Genomics (ACMG) recommended list of secondary findings and the Recommended Uniform Screening Panel (RUSP) [19, 20]. One challenge of a gene-based approach is that a gene can have more than one disease association. This can be due to differences in molecular mechanism such as recessive loss-of-function versus dominant gain-of-function, or due to alterations of certain regions of the encoded protein that have specific functional roles. In other cases, there are no true genotype-phenotype correlations and the ultimate clinical presentation cannot be accurately predicted based on molecular analysis. For some, the panel of genes may not specify which of the disease phenotypes is intended to be interrogated by the panel, in which case it is not clear if the lab would only return variants associated with the more actionable phenotype or all pathogenic or likely pathogenic variants in the gene regardless of phenotypic association. Whenever this situation occurred, we generated a SQM score for each disease phenotype separately, thus allowing us to reflect a single score for the most actionable clinical phenotype. Of note, however, when clinical labs are not transparent about which gene-disease association is being interrogated in a given test, it raises the concern that results being returned could reflect a condition that is much less actionable than would be expected in a newborn screening setting.

While the aggregate actionability of each of the panels was high (median SQM scores of 11 or 12), each panel included genes that fell below our threshold for definite actionability and would not have been included in our Category 1 list. Examples include *NF2* and *SERPINA1* genes with associated conditions (neurofibromatosis type 2 and emphysema-cirrhosis due to alpha-1-antitrypsin deficiency, respectively) and interventions that were considered actionable by the scoring committee, but onset of disease outcomes of interest (acoustic neuromas and meningiomas for neurofibromatosis type 2 and emphysema and cirrhosis for alpha-1-antitrypsin deficiency) more commonly occurred in adulthood [39, 40]. Thus, the genes were placed in ASQM Category 3. PerkinElmer’s panel also included *HGD*, *NAGA*, and *PRNP* that were placed in ASQM Category 4 or not eligible for return to parents in childhood. *HGD* is associated with alkaptonuria which can result in arthritis in adulthood and does not have an efficacious intervention that ameliorates symptoms [41]. *NAGA* and *PRNP* are associated with Kanzaki disease/Schindler disease type II and prion diseases, respectively, all of which have neurologic manifestations occurring mainly in adulthood and for which there are no effective interventions that can improve or prevent symptoms of disease though potential therapies are being studied [42, 43]. Transparency in these types of offerings is important since there may be significant clinical and psychosocial consequences of learning about a condition that is less actionable for infants and children than may have been perceived in the marketing of a product. Thus, we recommend that genomic screening of newborns be confined only to the most actionable childhood-onset conditions.

*MCCC1*, *MCCC2*, *PCBD1*, *DLD*, and *HPD* are examples of genes given low ASQM knowledge base criterion scores of 1 or 0 that were placed in ASQM Category 2. These gene-disease pairs also had a score of 0 for one or more of the other four criteria due to extremely limited evidence. *FMR1* associated with fragile X syndrome was included on the BabyGenes panel and placed in ASQM Category 2 by our group. Assuming that the sequencing technology used for the BabyGenes panel is capable of picking up the relevant triplet repeat expansion, evidence for the efficacy of early childhood intervention is still considered insufficient for routine inclusion at the present time. Of note, current research is underway to explore efficacy in this condition [44]. Therefore, evaluation over time is needed and actionability can change with new evidence for gene-disease pairs including *FMR1* and fragile X syndrome.

Furthermore, there are genes included on all four commercial laboratory panels that are associated with clinical phenotypes that have differing levels of actionability. Some of these differences relate to variable expressivity within a given disease spectrum (e.g., early onset versus later onset of symptoms in Citrullinemia Type II, or Gaucher disease) which may be difficult to tease apart simply based on the genetic variants identified [45, 46]. In other examples, differences in molecular mechanism should enable laboratories to predict which condition is more likely based on the variant(s) identified and report only those variants that are known to be implicated in the more actionable condition.

Lastly, downstream ramifications also threaten the benefit of actionable information and add to the many ethical, legal, and social issues raised by genomic screening in newborns and children. For example, it is unclear what follow-up steps will be recommended by labs or taken by providers, and whether uniform management programs for individuals identified through genomic screening will be available as they are for state-sponsored newborn screening. Cascade testing of family members would also be relevant for consideration by clinicians and policy-makers. In addition, the types of results that are eligible for disclosure should be clearly defined. For example, if carrier status were to be returned for recessive conditions, the vast majority of positive results would be heterozygous variants indicating carrier status (based simply on Hardy-Weinberg proportions), which would derail the intent of genomic screening to identify rare individuals with clinically actionable molecular findings. Clarification and resolution of these points are needed for specific genomic screening offerings as well as in the broader context of policy recommendations.

## Conclusions

Genomic screening in neonates, infants, and children presents an opportunity to ameliorate disease outcomes and thereby improve public health [47]. It is critical, however, to proceed in an evidence-based way with transparency about what conditions are being evaluated and what the results mean with respect to the positive predictive value and negative predictive value of such testing. Public health improvements could likely occur when genomic screening moves into the newborn screening public health system. However, the current newborn screening system does not have the capacity to take on this type of testing and the follow-up it would require, and commercial laboratory genomic screening options are currently separate from this system. Early adoption of such technology is likely to take place as a supplemental NBS pursued by families with greater awareness of genomic technology, high information seeking preferences, and the means to pay for testing out-of-pocket. However, such selective uptake will limit insight into implementation strategies and health outcomes necessary to serve broad populations, and therefore truly impact public health, and could exacerbate disparities in health care.

In the future, we envision that genomic screening panels could be tailored to the child’s age in order to deliver timely and clinically relevant genomic information throughout pediatric well-child care [48]. However, the clinical utility of this age-based genomic screening approach, and the barriers and facilitators of its implementation in a diverse population, need to be evaluated through rigorous studies. Additionally, plans for comprehensive, cohesive follow-up care through the lifespan that consider future management and a “medical home” for coordinating care are currently lacking for many conditions included on genomic screening panels even though sequencing technologies may be ready. While many challenges remain, efforts focused on disparate populations and tailored information based on age will move genomic screening closer to its implementation as a part of precision public health.

## Supplementary Information

**Additional file 1. **An excel spreadsheet containing four tabs with Supplemental Tables S1-S4. **Table S1.** provides a list of the 74 gene-disease pairs included on all commercial panels with ASQM criteria scores, total score, and designated category for each gene-disease pair. **Table S2.** lists 88 additional gene-disease pairs that were scored for this manuscript following the publication by Milko et al. [19], including individual ASQM criteria scores, total score, and designated category for each gene-disease pair. **Table S3.** lists the 77 genes that appeared on one or more commercial laboratory NBS panels, but were designated by our group as ASQM Category 2 because they have childhood-onset but lower or no actionability. **Table S4.** includes 249 genes that were designated by our group as ASQM Category 1 due to childhood onset and high actionability, but were not listed on any of the four commercial lab NBS panels.

## Data Availability

All data generated or analyzed during this study are included in this published article, its supplementary information files, and in supplementary information files in Milko et al. 2019 [19].

## Abbreviations

ACMG: American College of Medical Genetics and Genomics
ASQM: Age-based, semi-quantitative metric
CF: Cystic fibrosis
GTR: Genetic Testing Registry
NBS: Newborn screening
NGS: Next-generation sequencing
NIH: National Institutes of Health
RUSP: Recommended Uniform Screening Panel
SMA: Spinal muscular atrophy
SQM: Semi-quantitative metric
